# Supplementary material for: Analytical Techniques Applied to the Study of Industrial Archaeology Heritage: The Case of Plaiko Zubixe Footbridge
Source: Molecules. 2022 Jun 4;27(11):3609. doi: 10.3390/molecules27113609 (PMC9182420; doi:10.3390/molecules27113609)
Supplement: Supplementary file 1 [file molecules-27-03609-s001.zip › molecules-1739812-supplementary.pdf]

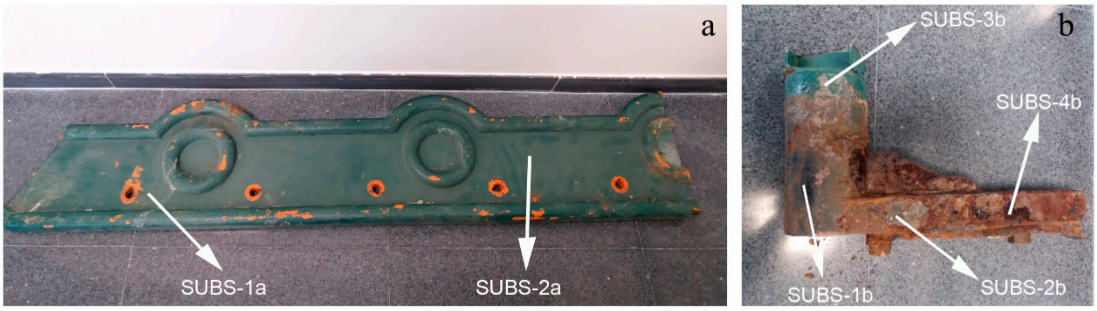

Figure S1. Parts of the rotating bridge from which the samples analysed were extracted.

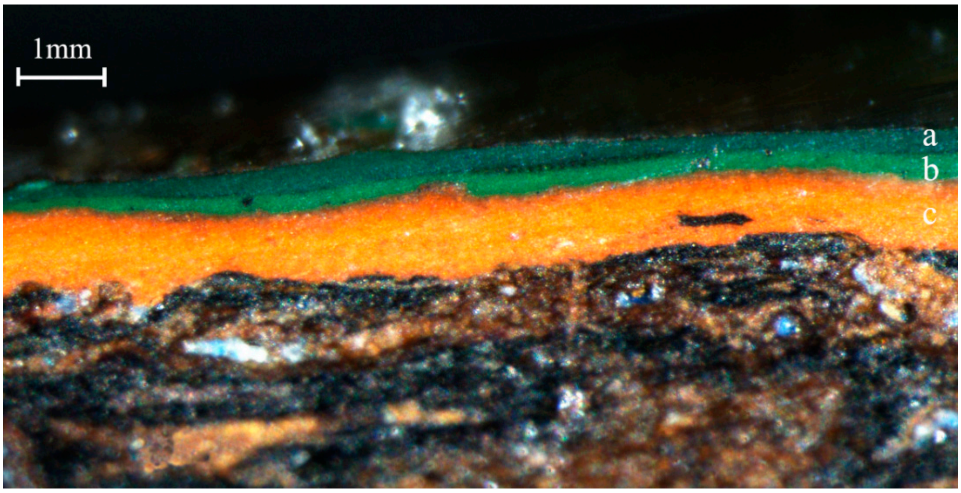

Figure S2. Image of the cross-section (sample SUBS-1a) from the piece that belongs to the lower part of the railing showing the three paintings layers and the iron support.

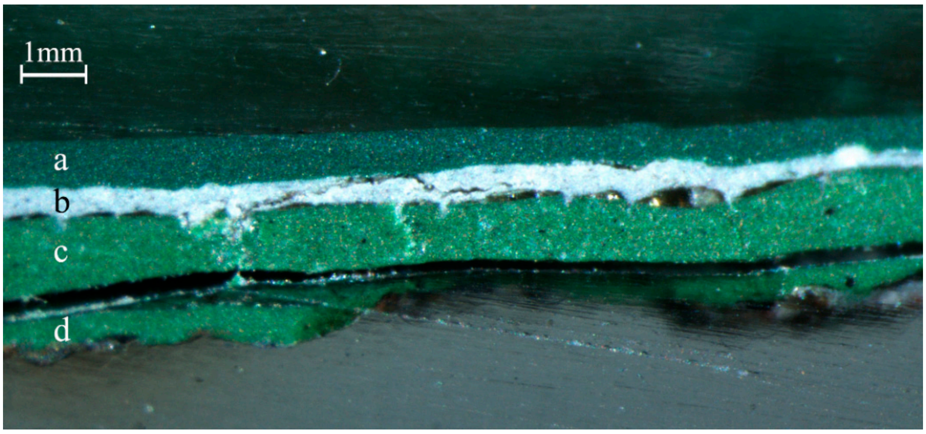

Figure S3. Image with a stereoscopic microscope of the sample collected in a green area.

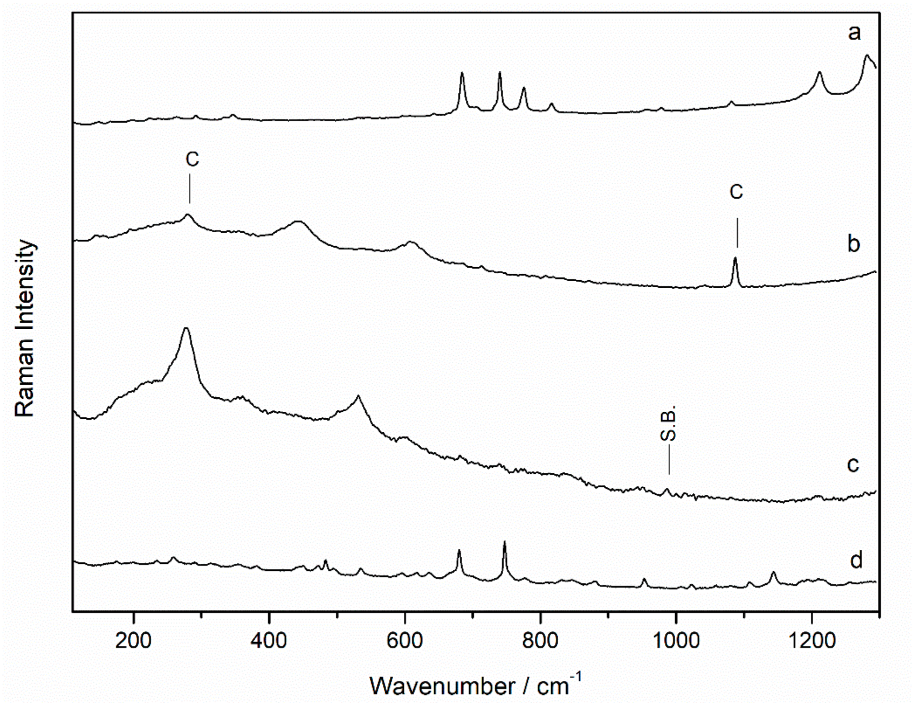

Figure S4. Raman spectra of the compounds found in the subsample collected from a green area: phthalocyanine green (a), rutile plus calcium carbonate (C) (b), Prussian blue plus barium sulfate (SB) (c) and phthalocyanine blue (d).

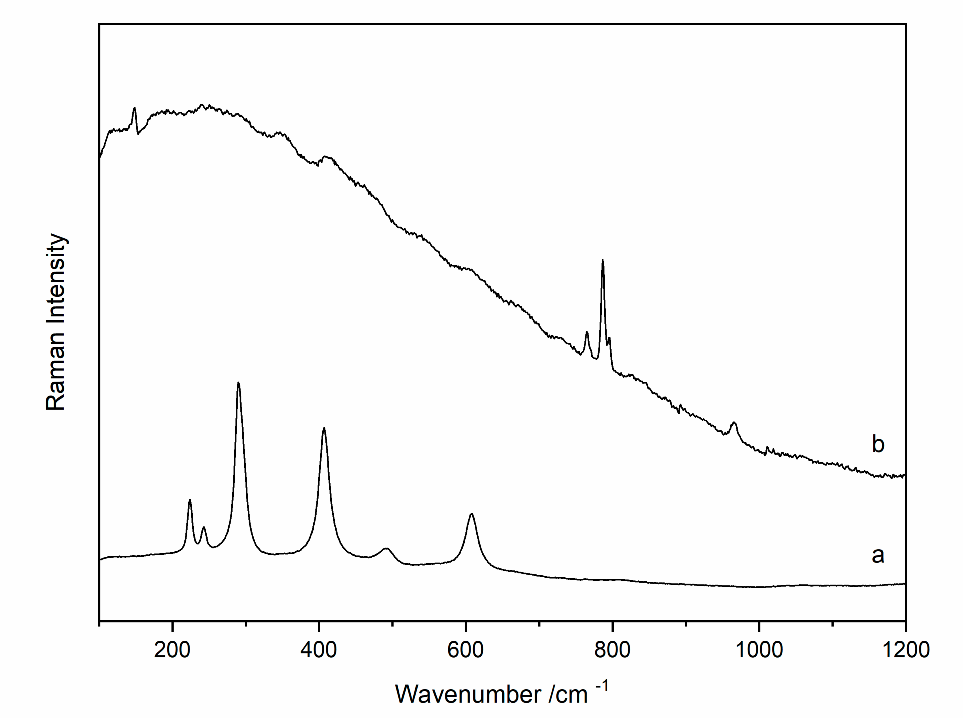

Figure S5. Raman spectra of hematite (a) and of the silicon-carbon compound (b).

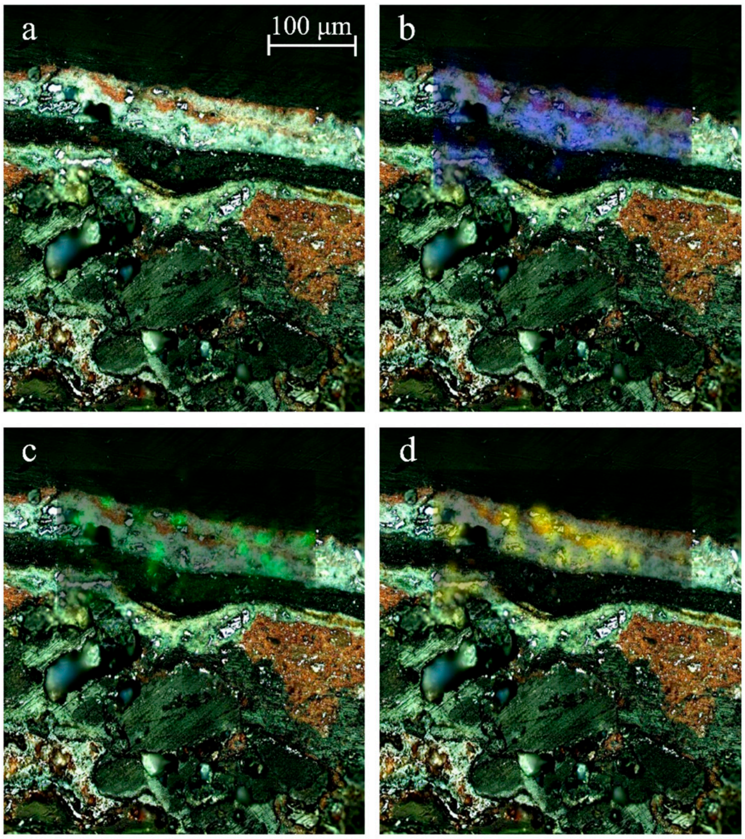

Figure S6. Raman images show the distribution of hematite (b), a silicon and carbon material (c) and barium sulfate (d).

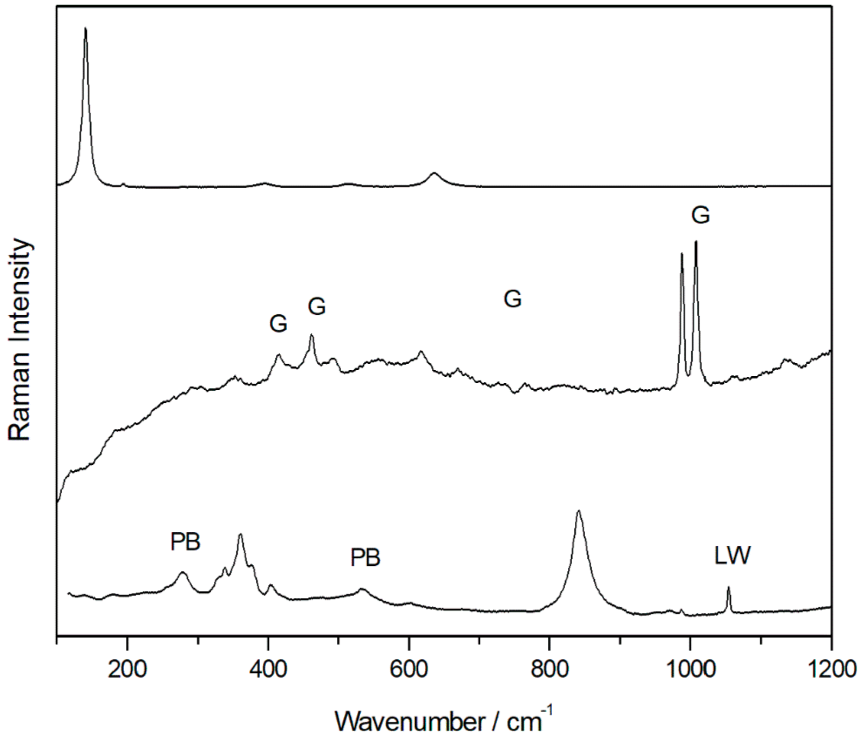

Figure S7. Raman spectra of barium lead chromate with Prusian blue (PB) and lead white (LW) sulfate plus gypsum (G) (b) and anatase (c) from the sample SUBS-3b.

Table S1. Subsamples collected from each piece of the rotating bridge and techniques employed for their characterization.

Piece	Subsample 1	Subsample 2	Subsample 3	Subsample 3
**P1a**	SUBS-1a	SUBS-2a		
Techniques	μ-Raman	μ-Raman		
**P1b**	SUBS-1b	SUBS-2b	SUBS-3b	SUBS-4b
Techniques	μ-Raman	μ-Raman	μ-Raman μ-EDXRF	μ-Raman,
